# Cancer-Related Information Seeking and Scanning Behaviors among Older Chinese Adults: Examining the Roles of Fatalistic Beliefs and Fear

**DOI:** 10.3390/geriatrics2040038

**Published:** 2017-12-03

**Authors:** Doris Y. P. Leung, Twiggy T. Chow, Eliza M. L. Wong

**Affiliations:** 1 The Nethersole School of Nursing, The Chinese University of Hong Kong, Hong Kong, China; 2 Sau Po Center on Ageing, University of Hong Kong, Hong Kong, China; twiggychow@yahoo.com.hk; 3 School of Nursing, Hong Kong Polytechnic University, Hong Kong, China; eliza.wong@polyu.edu.hk

**Keywords:** cancer information, seeking and scanning behaviors, fatalistic beliefs, cancer fear, aged

## Abstract

Effective communication in health information plays an important role in health promotion and cancer prevention. Cancer-related information acquisition can happen via active and purposeful seeking, but may also happen less purposely via the routine use of media and interactions with other people (called scanning). We examined seeking and scanning behaviors regarding cancer prevention in older Chinese adults, identified commonly used sources of information of such behaviors, and examined their associations with fatalistic beliefs and cancer fear. A convenience sample of 224 community-dwelling adults aged ≥60 were recruited between May and July in 2013 in Hong Kong. Results suggested that cancer information scanning (79.5%) was more common than information seeking (30.4%) among our participants. Health professional was the most popular source for both scanning (78.7%) and seeking (58.8%) behaviors regarding cancer information. Fatalistic beliefs was significantly and negatively associated with seeking behaviors (OR = 0.50) but not scanning behaviors, and cancer fear showed no relationship with either behavior. This study shows that the cancer information seeking and scanning behaviors were still suboptimal in this age group and adds to the knowledge regarding the associations between fatalistic beliefs and fear with cancer information seeking and scanning behaviors among older Chinese adults.

## 1. Introduction

Cancer burden continues to rise globally, especially in the elderly as incidence and mortality rates increase with age for most cancers [1]. According to the World Health Organization [2], over 14 million new cancer cases were diagnosed worldwide in 2012, and the number of new cases is expected to rise by about 70% over the next two decades. In China, it was estimated that the incidence rates of lung, colorectal, and prostate cancers will continue to rise even after adjusting for the change in the age structure in urban regions [3]. Although many cancer prevention interventions have been implemented, many older people still engage in cancer-related risky behaviors and do not take cancer preventive measures as recommended. Reports have also demonstrated that older people are more likely to be diagnosed with cancer via an emergency hospital visit rather than recognizing symptoms and actively seeking medical support at an early stage [4]. The increasing number of cancer patients and survivors with long lifespan and the high cost of cancer treatments have also shifted the cancer care from the hospital to the community, leading to an unprecedented dependence on carers’ support for high-quality care.

Effective communication in health information plays an important role in health promotion and cancer prevention. In the age of information technology, an abundant amount of health-related information is available through various media, including printed materials, TV, radios, health minutes, health talks, health-related websites, and individuals’ social networks. Recently, researchers have identified two distinct forms of behaviors in health communication: information seeking and scanning behaviors. In the literature [5,6,7,8], information seeking behavior refers to active and purposeful seeking of health information, while information scanning behavior refers to “information acquisition that occurs within routine patterns of exposure to mediated and interpersonal sources that can be recalled within a minimal prompt”, which is of less active efforts. One example of scanning behaviors includes the case where an individual pays a usual visit to the social center and hears a promotion of cancer screening and then their friends within the social network share cancer-related information and experience. Using the 2005 Health Information National Trends Survey, it was reported that scanning is more frequent than seeking in gaining health information, including colorectal cancer-related information [8].

Previous studies have shown that information seeking and scanning behaviors have been associated with cancer fear and fatalistic beliefs. One study reported that cancer fear related positively with cancer information avoidance [9], and another study found that individuals who never speak with family/friends about health were more likely to believe that colon cancer risk is not modifiable, while those provided with health information were less likely to believe that skin cancer risk is not modifiable [10]. However, previous studies have suggested that fatalism may be culture-specific, in that Chinese people tend to have a view of fatalistic voluntarism which refers to “a combination of efforts to change the situations and fatalistic acceptance of the way things are” [11,12]. We also found that the pessimism component in fatalism might not be applicable to the Chinese population [13]. In addition, Chinese people may place a great emphasis on conformity [14], and they may be more likely to obtain information via scanning and make their decisions based on interpersonal sources of information. While local studies have been focused on active searching for health information in the general population [15,16] and among older adults [17,18,19], no statistics on the seeking and scanning behaviors and the major sources in obtaining cancer health-related information among older Chinese adults are currently available. This study thus aims to describe the proportion of the exposure to cancer-related information via the use of seeking and scanning behaviors among older Chinese adults, in order to identify commonly used sources of information of both behaviors, and to examine their associations with fatalistic beliefs and cancer fear separately. 

## 2. Materials and Methods 

### 2.1. Study Design and Participants

This is a cross-sectional survey using a standardized and structured questionnaire conducted between May and July 2013 in Hong Kong. Community-dwelling older adults aged 60 or above visiting three selected social centers for the elderly of a non-government organization (NGO) were invited to participate in the study. Participants were excluded if they (1) were currently diagnosed with a mental disease or a history of cancer, had a severe cognitive impairment (i.e., had a score in Mini-Mental State Examination (MMSE) <18) [20], (2) could not communicate in Cantonese or other dialects of Chinese, or (3) suffered from hearing or visual impairment. A team of four-to-five student helpers with training in communication with older adults screened for subject eligibility at the centers. After obtaining written consent from eligible subjects, the student helpers administered the survey instrument face-to-face by asking the respondents the questions at the center premises. A total of 260 older adults completed the questionnaires. Data from 36 participants with missing data in the study variables were excluded, resulting in a final sample of 224 (86.2%) for the current analysis. The study was approved by the Survey and Behavioural Research Ethics Committee of the Chinese University of Hong Kong and from the participating organizations. 

### 2.2. Measures

Cancer-related information seeking and scanning behaviors: We used the scale developed by Niederdeppe et al. and then validated by Kelly et al. to collect data about respondents’ sought and scanned information exposure about three prevention and three scanning behaviors, including exercise, fruit and vegetable consumption, weight-loss attempt, colonoscopy, the prostate test (for men only), and mammography (for women only) [5,6,7]. For information-seeking behaviors, participants were asked whether they had taken actions in seeking information on each of the six topics actively in the past 12 months. For those responding “Yes” to the question, they were further asked about the sources they had sought for the information from six sources, including (i) medical professionals; (ii) family, friends, or coworkers; (iii) television or radio; (iv) newspapers, magazines, or newsletters; (v) the Internet; and (vi) other sources. Similar for information scanning behaviors, participants were asked whether they had come across information on the topic in the past 12 months, and then this was followed by the source questions. An overall index for information seeking and scanning behaviors was respectively created by summing the number of sources of information for the specific topics, each with a possible range of 0–30 (up to six sources for a total of five topics per gender). Participants scoring 0 in information seeking behaviors were then classified as non-seekers, while those scoring 0 in information scanning behaviors were classified as non-scanners. The instrument was validated with a nationally-representative sample of 2489 adults in the US with good face, convergent and discriminant validity, and applied successfully in a population-based study [6,7]. 

Fatalistic beliefs were measured using a 15-item fatalism scale adapted from the original 20-item scale developed by Shen and colleagues [21]. The revised scale consists of three dimensions of disease-specific predetermination (six items), general predetermination (four items), and luck (five items) [13]. Respondents were asked to rate the items using a five-point Likert scale ranging from 1 (Strongly disagree) to 5 (Strongly agree). A composite score of fatalism was created by summing all 15 items. The scale scores can range from 15 to 75, with a high score indicating higher level in fatalism. It has been reported to have good psychometric properties in local elderly samples [22,23]. The scale also has a Cronbach’s alpha of 0.941 in the current sample.

Cancer fear was measured by means of eight items from the Breast Cancer Fear Questionnaire, originally developed by Champion and colleagues in 2004 [24], with the wording altered to refer to cancer in general. Responses were gathered with a five-point Likert scale ranging from 1 (strongly disagree) to 5 (strongly agree). The scale scores can range from 8 to 40, with higher scores indicating higher level of fear. The alternative version of the scale measuring cancer in general has been shown to have a high reliability (α = 0.91) in an older adult sample [9]. The scale has also been modified to measure fear related to colorectal cancer in older Chinese adults [25]. Cronbach’s alpha value of the scale was 0.974 in the current sample.

We also collected demographic variables including age, gender, education level, marital status, financial status, religion, cognitive functioning, and presence of chronic diseases and family history of any types of cancer. Cognitive functioning was measured by the 11-item Mini-Mental State Examination [26]. The Cantonese version of the MMSE showed good reliability and validity among Hong Kong elders [20]. 

### 2.3. Data Analysis

Proportions for categorical variables and means and standard deviations for continuous variables were employed to summarize the levels of fatalistic beliefs, cancer fear, cancer-related information seeking and scanning behaviors, and the sample characteristics. Two sets of logistic regression analysis with a hierarchical block design were performed to examine the individual effects of fatalistic beliefs and cancer fear on the likelihood of performing information seeking and scanning behaviors separately. The first step of the hierarchical logistic regression included fatalistic beliefs and cancer fear as the independent variables in the model, and the second step added all the demographic characteristics (with educational level and household income treated as continuous variables) to the model in order to control their potential effects on the dependent variable. Odds ratios and the corresponding 95% confidence intervals were reported. All analyses were performed with SPSS version 22.0 (IBM Corporation; Armonk, NY, USA) (city, state abbreviation if USA, country), and statistical significance was set at 5%. 

## 3. Results

Among the 224 respondents, a majority were female (60.3%), their mean age was 77.2 (SD = 7.0), about half were married, more than a half had no formal or primary education, and 18.3% had a family member having cancer. Their mean household income was low, and their mean MMSE score was 26.4 (SD = 3.3), which was very high. During the past 12 months, a minority of the participants reported that they had actively sought (30.4%) while a majority had scanned (79.5%) cancer-related information. The participants had a mean level of fatalism close to the middle point, while a mean level of cancer fear on the lower side (Table 1). 

**Figure 1:**
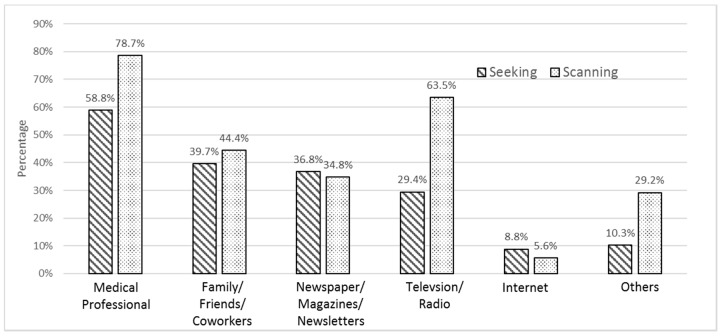
Proportions of cancer-related information seeking and scanning behaviors by source.

Figure 1 displays the sources of information seeking and scanning behaviors among the participants. Among those who had engaged in seeking behaviors (*n* = 68), healthcare professional was the most popular source (58.8%), family/friends/coworkers (39.7%) the second, and newspaper/magazines/newsletters (36.8%) the third. Among those who had engaged in scanning behaviors (*n* = 178), healthcare professional was also the most popular source (78.7%), followed by television/radio (63.5%), and then family/friends/coworkers (44.4%). Internet was the least favorable source for both behaviors. 

Table 2 presents data of the hierarchical logistic regression for cancer-related information seeking behaviors. The results show that fatalistic beliefs remains negatively and statistically significant while cancer fear becomes non-significant after controlling the effects of the demographic variables. Among the demographic variables, being married is also positively and significantly associated with the seeking behaviors. This indicates that participants who had a lower level in fatalistic beliefs and being married were more likely to actively engage in the behaviors of seeking cancer-related information. 

Table 3 presents logistic regression models for cancer-related information scanning behaviors. It reveals that both fatalistic beliefs and cancer fear were not associated with the scanning behaviors. On the other hand, three demographic variables were significant associated factors: no family member had cancer was negatively associated, while being female and having a higher MMSE score were positively associated with the likelihood of having scanning behaviors. 

## 4. Discussion

The present study was designed to explore the level of cancer-related information acquisition via purposely seeking and less-actively scanning in older Chinese adults. Consistent with the population-based study in the US [14], we also found that a substantial difference in the two studied information acquisition behaviors, with scanning being much more reported than seeking among our participants. The current finding adds further support to the claim that more people are scanning than seeking for health-related information, including older Chinese adults. This highlights the importance of measuring scanning behaviors in health communication in research.

We found that fatalistic beliefs were associated with seeking but not scanning behaviors. Previous studies also reported no significant relationships of fatalistic beliefs with participation in colorectal cancer screening in older Chinese adults in Hong Kong [27], and no relationship between fatalism and medical screening in Chinese immigrants in Australia [28]. On the other hand, another study reported that fatalism related significantly with a lower adherence rate to colorectal cancer screening in Asian Americans [29]. The inconsistent finding might be due to the use of the Powe Fatalism Inventory that might not be applicable to the Chinese heritage [30] and the fact that the Asian group included people originating from the Asian region with different cultures in the latter study. These findings may suggest that Chinese people may have a distinct mechanism on how fatalistic beliefs influences cancer preventive measures. In particular, it may be possible that fatalistic beliefs are a moderating factor that influences the participation in cancer screening indirectly through active information seeking behaviors, rather than being an independent predictor of cancer screening participation. 

In line with our previous study in colorectal cancer screening [27], this study also found an insignificant result of cancer fear on both seeking and scanning behaviors to acquire cancer-related information. While contradictory to findings from the review on older adults which had shown that fear may either be a facilitator of or a barrier to participation in colorectal cancer screening [31], a series of studies on older Chinese adults suggest that there may be a culture-specific pattern of the relationship between fear and cancer-related preventive behaviors [13,22,25,27]. Alternatively, there are some moderating factors such as fatalistic beliefs or mediating factors such as self-efficacy acting on such a relationship among Chinese people. Future studies should further examine the role of fatalistic beliefs on health preventive measures in general and cancer-related preventive measures in particular.

Our study also found that the participants relied heavily upon medical sources and television/radio for both seeking and scanning behaviors. Previous studies had reported that older adults were inclined to rely upon healthcare professionals for medical information [32]. This is also consistent with the most common methods for health promotion among older adults in Hong Kong, which are health talks at general outpatient clinics and inviting healthcare professionals to present their health advice on television or radio [18]. Although the Internet was not a popular information source for both scanning and seeking, as many older people in Hong Kong were not frequent Internet users, it has a great potential as an effective media for health communication. Indeed, it was estimated that only 13.1% of the Hong Kong adults aged 65 years or above had used the Internet in the past 12 months in 2012, but the trend of internet users is increasing due to the advance in technology—especially the development of the web and smart phone [33]. With the staff shortage and the huge demand for services in the busy clinical setting, these findings highlight the importance of exploring the potential usage of the Internet for health information provision or health promotion, such as providing a health Web-navigating workshop for older adults delivered by professionals to equip them with literacy in both IT and health [18].

The current study has several limitations. We recruited participants from NGOs using a convenience sampling, which limits the generalizability of the study findings. In particular, the proportion of scanning behaviors might have been over-estimated, as the participants might have engaged in scanning behaviors because they might have already received some health talks or health information from the NGO they visited. Older adults who did not visit NGOs are unlikely to receive this medical information via this particular source. The findings are also subject to report-bias, especially for older adults. The measurement tool for seeking and scanning behaviors used in this study might not be accurate enough, as it only captured the total number of sources sought and scanned from the six cancer preventive behavior topics that could not reflect the depth of seeking and scanning which occurred [34]. In addition, we have only included a few variables in the logistic regression models. It is possible that we might have omitted some important factors of the two behaviors, such as self-efficacy and cues to actions [27], so as to improve the predictive power of the models. Furthermore, the associations observed could not be regarded as causal due to the use of a cross-sectional design, and the findings should be further examined in longitudinal studies to establish the temporal validity of any associations found.

## 5. Conclusions

The study shows that the proportions of cancer information seeking and scanning behaviors were suboptimal in this older adult sample, suggesting a need to provide support for further promotion of effective health communication in this age group. Although the Internet was the least commonly used source for both behaviors, its potential usage as an effective channel for health communication should not be undermined. This study also adds to the knowledge regarding the factors associated with these two types of behaviors for cancer information acquisition: fatalistic beliefs were associated with seeking behaviors but not scanning behaviors, while cancer fear did not associate with either behavior. More research is needed to better understand the mechanisms by which fatalistic beliefs affect cancer preventive behaviors, if any, among older Chinese adults. 

## Figures and Tables

**Table 1:** Sample characteristics and proportions of cancer-related information seeking and scanning behaviors (*n* = 224).

	*n* (%)	Mean ± SD
**Deomgraphic characteristics**		
Female	135 (60.3)	
Married	114 (50.9)	
Educational level		
No formal education	58 (25.9)	
Primary education	95 (42.4)	
Secondary 1–Secondary 3	26 (11.6)	
Secondary 4–Secindary 5	24 (10.7)	
Secondary 6–Secondary 7	8 (3.6)	
Tertiary education	12 (5.4)	
Above Tertiary education	1 (0.4)	
No family member had cancer	183 (81.7)	
Age		77.2 ± 7.0
Numer of chronic diseases		1.2 ± 1.2
Household income ^1^		1.6 ± 1.5
MMSE score		26.4 ± 3.3
Fatalistic beliefs		43.5 ± 11.3
Cancer fear		19.7 ± 4.8
**Outcome variables**		
Cancer-related information seeking behaviors	68 (30.4)	
Cancer-related information scanning behaviors	178 (79.5)	

^1^ Response for Household Income: 1 = <HKD5000; 2 = HKD5000–HKD9999; 3 = HKD10,000–HKD14,999, 4 = HKD15,000–HKD19,999, 5 = HKD20,000–HKD24,999, 6 = HKD25,000–HKD29,999, 7 = HKD30,000–HKD39,999, 8 = ≥40,000.

**Table 2:** Outcome of hierarchical regression analysis of cancer-related information seeking behaviors. MMSE: Mini-Mental State Examination.

	Model 1 Adjusted Odds Ratio (95% CI)	Model 2 Adjusted Odds Ratio (95% CI)
Fatalistic beliefs	0.40 (0.25–0.62)	0.50 (0.30–0.84)
Cancer fear	2.01 (1.18–3.43)	1.76 (0.97–3.21)
Age		0.97 (0.93–1.02)
Female		1.85 (0.85–4.04)
Married		2.24 (1.09–4.62)
Educational level		1.14 (0.87–1.50)
Household income		1.01 (0.81–1.27)
No family member has cancer		0.62 (0.27–1.41)
Number of chronic diseases		1.01 (0.76–1.35)
MMSE score		1.13 (1.00–1.28)
Nagelkerke R^2^	0.124	0.253

**Table 3:** Outcome of hierarchical regression analysis of cancer-related information scanning behaviors.

	Model 1 Adjusted Odds Ratio (95% CI)	Model 2 Adjusted Odds Ratio (95% CI)
Fatalistic beliefs	0.74 (0.47–1.15)	0.98 (0.57–1.68)
Cancer fear	0.92 (0.52–1.61)	0.76 (0.41–1.40)
Age		0.98 (0.92–1.03)
Female		4.24 (1.77–10.12)
Married		1.28 (0.57–2.86)
Educational level		1.36 (0.93–2.00)
Household income		0.97 (0.68–1.37)
No family member has cancer		0.24 (0.06–0.94)
Number of chronic diseases		0.86 (0.63–1.18)
MMSE score		1.19 (1.05–1.34)
Nagelkerke R^2^	0.015	0.233
